# Spatial Learning and Memory in Barnes Maze Test and Synaptic Potentiation in Schaffer Collateral-CA1 Synapses of Dorsal Hippocampus in Freely Moving Rats

**DOI:** 10.32598/bcn.9.10.330

**Published:** 2019-09-01

**Authors:** Azam Sadeghian, Yaghoub Fathollahi, Mohammad Javan, Amir Shojaei, Nastaran Kosarmadar, Mahmoud Rezaei, Javad Mirnajafi-Zadeh

**Affiliations:** 1. Department of Physiology, Faculty of Medical Sciences, Tarbiat Modares University, Tehran, Iran.; 2. Department of Brain and Cognitive Sciences, Cell Science Research Center, Royan Institute for Stem Cell Biology and Technology, ACECR, Tehran, Iran.

**Keywords:** Synaptic plasticity, Hippocampus, Barnes maze test, Spatial memory

## Abstract

**Introduction:**

Synaptic plasticity has been suggested as the primary physiological mechanism underlying memory formation. Many experimental approaches have been used to investigate whether the mechanisms underlying Long-Term Potentiation (LTP) are activated during learning. Nevertheless, little evidence states that hippocampal-dependent learning triggers synaptic plasticity. In this study, we investigated if learning and memory in the Barnes maze test are accompanied by the occurrence of LTP in Schaffer collateral to CA1 synapses in freely moving rats.

**Methods:**

The rats were implanted with a recording electrode in stratum radiatum and stimulating electrodes in Schaffer collaterals of the CA1 region in the dorsal hippocampus of the right hemisphere. Following the recovery period of at least 10 days, field potentials were recorded in freely moving animals before and after training them in Barnes maze as a hippocampal-dependent spatial learning and memory test. The slope of extracellular field Excitatory Postsynaptic Potentials (fEPSPs) was measured before and after the Barnes maze test.

**Results:**

The results showed that the fEPSP slope did not change after learning and memory in the Barnes maze test, and this spatial learning did not result in a change in synaptic potentiation in the CA1 region of the hippocampus.

**Conclusion:**

Spatial learning and memory in the Barnes maze test are not accompanied by LTP induction in Schaffer collateral-CA1 synapses.

## Highlights

- Spatial learning and memory in the Barnes maze test could not change the slope of extracellular field excitatory postsynaptic potentials in rats;
- Spatial learning and memory in the Barnes maze test did not result in a change in synaptic potentiation in the CA1 region of the hippocampus;
- Spatial learning and memory in the Barnes maze test are not accompanied by long-term potentiation induction in Schaffer collateral-CA1 synapses.

## Plain Language Summary

Nowadays, many experimental approaches investigate whether the mechanisms underlying Long-Term Potentiation (LTP) are activated during learning. It has also been reported that learning and memory facilitate plasticity, which shows the association between the different forms of synaptic plasticity and the different components of spatial memory. Although different types of synaptic plasticity such as LTP or long-term depression affect learning and memory, few evidences have shown changes in synaptic plasticity in the CA1 region during hippocampal-dependent tasks. Therefore, in the present study, we tried to address if hippocampal-dependent spatial learning task such as Barnes maze test leads to synaptic plasticity in the CA1 of the dorsal hippocampus. The extracellular field Excitatory Post-Synaptic Potentials (fEPSP) slope was used as a measure of excitatory synaptic transmission in the CA1 region in freely behaving rats. Based on the results, there were no substantial changes in the strength of Schaffer collaterals-CA1 synapses in the dorsal hippocampus. Field potential recordings before and after the Barnes maze test revealed that this hippocampal-dependent spatial learning task did not significantly change synaptic efficacy in the CA1 area of the dorsal hippocampus. Overall, our study did not support the idea that plasticity in Schaffer collaterals-CA1 synapses of the dorsal hippocampus is triggered by spatial learning events.

## 1. Introduction

The phenomenon of plasticity discovered over 40 years ago in the hippocampus (Lømo, 2003). Since then, many researchers assume that Long-Term Potentiation (LTP) and Long-Term Depression (LTD) in particular brain regions are the fundamental physiological mechanisms underlying memory function. This conclusion was based on the properties of these phenomena that can change the synaptic strength and the role of these brain regions in learning and memory. Recently, Nabavi et al. have reported a causal link between synaptic plasticity and memory. They showed that fear conditioning memory could be inactivated and reactivated by optogenetically-delivered LTD and LTP in the amygdala, respectively (Nabavi et al., 2014). However, direct evidence proves that hippocampal LTP is induced by learning.

Nowadays, many experimental approaches investigate whether the mechanisms underlying LTP are activated during learning. Some researchers tried to find the correlation between learning task and cellular and molecular factors involved in LTP. For example, it has been shown that the inhibitory avoidance task (a hippocampal-dependent task) causes the delivery of α-amino-3-hydroxy-5-methyl-4-isoxazolepropionic acid (AMPA) receptors to hippocampal synaptoneurosomes and phosphorylation of hippocampal glutamate receptors (Whitlock, Heynen, Shuler, & Bear, 2006). It has also been reported that learning and memory facilitate plasticity, which shows the association between the different forms of synaptic plasticity and the different components of spatial memory (Kemp & Manahan-Vaughan, 2007). In another experiment, it was demonstrated that an afferent stimulation pattern, which was subthreshold for the induction of plasticity, could lead to persistent synaptic potentiation for days or weeks when accompanied by a learning task (Kemp & Manahan-Vaughan, 2012).

Field potential recording is a suitable way to monitor the strength of the population of synapses. The laminated synaptic arrangement of the hippocampal formation allows the extracellular field Excitatory Postsynaptic Potentials (fEPSP) to be used as an index of the number of the excitatory synapses activated by a given afferent impulse volley. Several studies show changes in synaptic efficacy during behavioral tasks in the perforant path to dentate gyrus granule cell synapse (Doyere et al., 1995; Skelton, Scarth, Wilkie, Miller, & Phillips, 1987; Weisz, Clark, & Thompson, 1984) and Schaffer collateral to CA1 pyramidal cells (Whitlock et al., 2006). Although different types of synaptic plasticity such as LTP or LTD affect learning and memory, few documents demonstrate changes in synaptic plasticity in the CA1 region during hippocampal-dependent tasks. Therefore, in the present study, we tried to address if hippocampal-dependent spatial learning task such as Barnes maze test (Fox, Fan, LeVasseur, & Faden, 1998) leads to synaptic plasticity in the CA1 of the dorsal hippocampus, the area that is a site of robust synaptic plasticity (Taubenfeld et al., 2001) and involves in spatial memory formation (Eichenbaum, 1999; Morris, Garrud, Rawlins, & O’Keefe, 1982).

### Experimental procedures

#### Study Animals

Adult male Wistar rats (8–9 weeks old) obtained from the Pasteur Institute of Tehran, Iran. They were maintained in the animal cage at a constant temperature of 22°C±2°C on 12:12 light/dark cycle. Animals were individually housed and permitted free access to food and water. The experiment was done with the ethical guidelines set by the Ethics Committee of the School of Medical Sciences, Tarbiat Modares University. All of the experiments were done at the same time to avoid the bias of circadian rhythms.

#### Operation

Under 100 mg/kg ketamine (10%, Alfasan, The Netherlands) and 10 mg/kg xylazine (20%, Alfasan, The Netherlands) anesthesia, the animals underwent stereotaxic implantation with a bipolar stimulating electrode in the Schaffer collaterals (coordinates: A, −3.1 mm; L, 3.5 mm; and V, 2.5–3 below dura) and a monopolar recording electrode in the stratum radiatum (coordinates: A, −2.8 mm; L, 1.8 mm; and V 2.3–2.5 mm below dura) of the right hemisphere according to the atlas of Paxinos and Watson. Stimulating and recording electrodes (stainless, steel, Teflon coated, 127 μm in diameter, A.M. Systems, USA) and the reference electrode (connected to the skull by a miniature screw) were insulated except at their tips.

The recording and stimulating electrodes were lowered and adjusted to maximize the fEPSP amplitude in the stratum radiatum in response to Schaffer collateral stimulation. The selective stimulation of the Schaffer collateral fibers was confirmed by observing the paired-pulse facilitation in response to the inter-pulse interval of 70 ms. After the verification of the electrode locations, all electrodes were connected to pins of the lightweight multichannel miniature socket as a head-stage and fixed on the skull with dental acrylic. The animals were allowed 10 days for recovery from the operation before starting the experiments.

#### Field potential recording

After recovery, field potentials were recorded in the recording box in a Faraday cage. The head-stage of the rat was connected to a flexible shielded cable. The rat was allowed to move freely during recording in the recording box. The fEPSP slope was used as a measure of excitatory synaptic transmission in the CA1 region in freely behaving animals. Basal synaptic transmission was recorded via test-pulse stimulation of the Schaffer collaterals, using a stimulation intensity determined from an Input/Output (I/O) relationship that was obtained for each animal. The test-pulse intensity was determined as the intensity that elicits about 50% of the maximum fEPSP was observed in the I/O curve and the range of 50 μA to 800 μA. The paired-pulse index was also measured by applying the same stimulus at 30 ms inter-pulse interval both before and after the Barnes maze test. The ratio of fEPSP slope in response to the second pulse to fEPSP slope in response to the first pulse was calculated as the paired-pulse index.

Responses were evoked by stimulating at low-frequency (0.1 Hz) with a single monophasic square wave pulse of 0.1 ms stimulus duration. For each time point, 12 evoked responses were averaged. Evoked responses were low-pass filtered at 3 kHz, using a PC-based DATA acquisition system (D3111 Science Beam instrument Co., Iran) and digitized at 10 kHz sampling rate, using a custom-designed software, eTrace analysis (version 2 ScienceBeam instrument Co., Iran). They were averaged and continuously monitored and stored on disk.

#### Barnes maze test

The Barnes maze test was used for assessing hippocampal-dependent spatial learning and memory. In this test, we used a high (90 cm to the floor) black Plexiglass circular platform (120 cm in diameter), containing 18 uniform holes (9 cm in diameter) in its periphery. Small removable and black Plexiglass plates were put at the beneath of all holes, except one hole that was connected to a removable black escape box (30 cm long × 15 cm wide × 10 cm deep). A black-squared starting box (20 cm × 20 cm long × 25 cm high) was used to place the rats on the platform. Two proximal visual cues were placed in the room, 50 cm away from the platform.

We used a protocol based on 4 days of acquisition trials, followed by a probe trial on the last day to assess maze acquisition and memory retention. One day before the first trial, the rats underwent a habituation routine to let them get acquainted with the platform and the escape box. An acquisition trial consisted of placing a rat in the starting box for 60 seconds (to ensure the randomization of the relative position of the rat in its encountering to maze). Then, the box was raised, and an aversive stimulus (bright light) was switched on, and the rat was allowed to explore the maze freely for 180 seconds. The rats were tested with the escape box 4 times per day (15-minute intervals between trials) for 4 consecutive days. On the fifth day, the rats were submitted to a probe trial for 180 seconds on the maze without an escape box. To eliminate olfactory cues, the surfaces of the maze and box were cleaned with 70% ethylic alcohol solution after each trial.

The behavioral performances were recorded using a computer-linked video camera mounted above the platform. The measured behavioral parameters included A. Escape box latency evaluated as the time spent by rat since its release from the start box to its entrance to the escape box during an acquisition trial or its first exploration of the escape hole during probe test; B. Number of errors considered as the number of explorations of non-scape hole. Each exploration of an incorrect hole is counted an as error; C. Search strategy was defined as: 1- direct (moving either directly to target hole or to an adjacent hole before visiting the target), 2- serial (the first visit to the target hole was preceded by visits to at least two of the non-target holes in a serial manner), and 3- random (unordered and random search of the maze).

### Experimental design

Field potential recordings were done in each animal before starting the first acquisition trial of the Barnes maze test and after probe test. To confirm the stability of synaptic responses, the recording was done continuously for 20 minutes. Field potential parameters were measured only in the animals, showing a significant decrease in the latency to target hole and the number of errors (as indices of learning), and their search strategy changed mostly toward direct strategy.

### Statistical analysis

The obtained data were averaged and expressed as Mean and Standard Error of the Mean (SEM). For each time point during the experiment, mean and S.E.M. were calculated from the averaged data of 12 successive evoked responses. A mean value of responses at 20 time points on the starting day of Barnes trails was defined as the baseline (100%). The recorded data after the Barnes test were expressed as the percentage change from the baseline. One-way ANOVA was used to determine changes in Barnes maze acquisition parameters. The probability level interpreted as statistically significant when P<0.05. The paired t-test was used to compare the changes in synaptic plasticity before and after the Barnes test.

## 3. Results

### Spatial learning acquisition and retention

**Figure 1.**
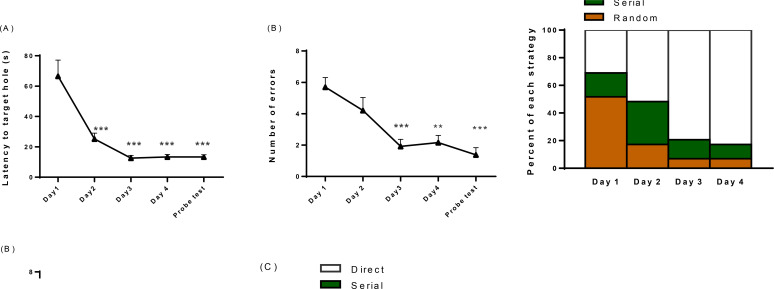
Learning and memory acquisition during 4 training days and probe test A. Latency to target hole; B. and the number of errors reduced significantly during training and probe test. C. The percent of strategies was also shifted from random toward direct. These parameters show significant improvement in learning and memory performance in the Barnes maze test. Data in A and B are presented as Mean±SEM. ** P<0.01 *** P<0.001 compared to day 1 (n = 8)

Following acquisition trails in 4 consecutive days, escape box latency decreased for all animals. One-way ANOVA showed a significant reduction in this parameter at days 2–4 compared to the first day (P<0.001; Figure 1A). We calculated the reduction in escape latency as the percentage of the latency of the first training day. The data showed 71.2%±7.4% performance in learning. In the probe test that the escape box was replaced by a small removable platform, the latency time of the animals to poke the location of the escape box was significantly shorter than the first day. Similarly, there was a significant decrease in the number of errors (60.9%±10.0%) during 4 days of acquisition trials and probe test (Figure 1B). Therefore, all animals showed a better performance in spatial learning acquisition and memory retention in Barnes maze.

Animals used 3 strategies to find the escape box. The random strategy refers to the lack of animal insight about the environment so that they randomly searched the maze. In serial strategy, the knowledge of animals about the environment and geometric location of the escape box increased. Finally, the animals learned the location of the escape box by using the spatial cues and used the direct strategy to find the scape box. Throughout the training, the random strategy dropped to negligible levels. The low percentage of random trials on the fourth day demonstrated that rats could learn the location of the escape box, using serial and direct strategies on the majority of trails during the last day of acquisition trials and probe tests (Figure 1C).

### Field potential recording from CA1 striatum radiatum

**Figure 2.**
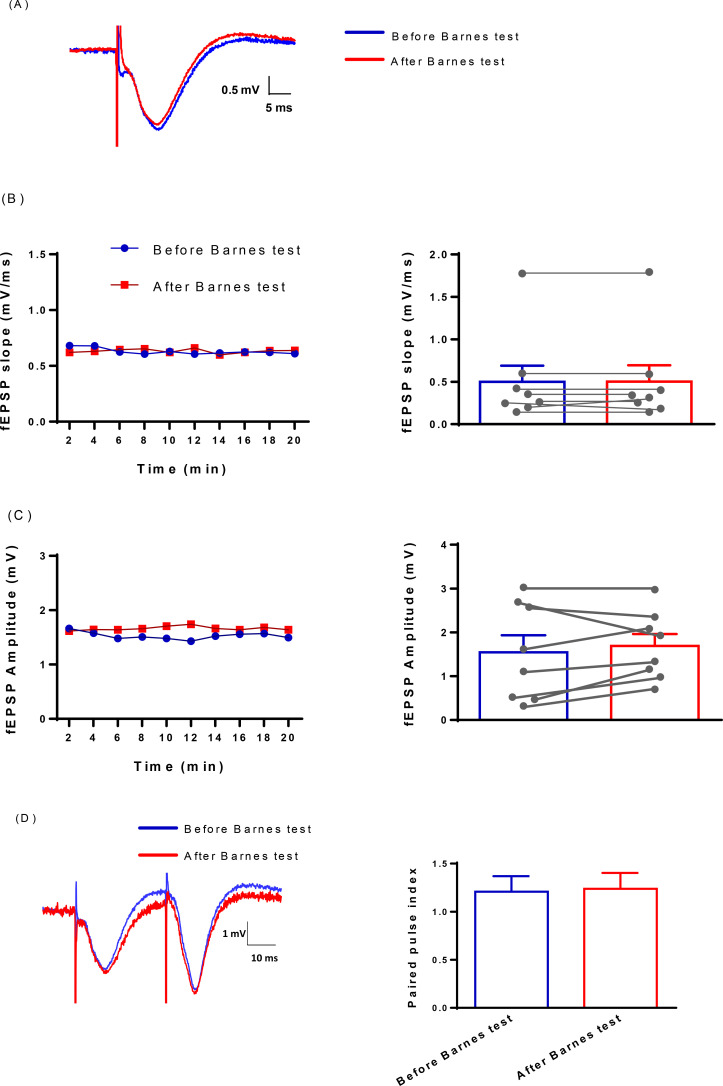
Evaluation of field potential recordings before and after the Barnes maze test A. Sample records showing field potentials recorded from apical dendrites of the CA1 region, following the stimulation of Schaffer collaterals; B and C. measuring the fEPSP slope and amplitude before and after the Barnes test showed no significant difference; D. Left: the paired-pulse recording samples at 30 ms inter-pulse interval before and after the Barnes test. Right: the paired-pulse index measured before and after the Barnes maze test had no significant difference.

Field potentials were recorded from the stratum radiatum of the hippocampal CA1 region before starting the Barnes test and after finishing the probe test. To have stable synaptic responses, field potentials were recorded for 20 minutes. Field potential parameters, i.e., elevated slope and amplitude, had no changes after Barnes maze acquisition (Figure 2). The paired-pulse index was also calculated at inter pulse-interval of 30 ms. There were no significant changes in the paired-pulse index before and after the Barnes test (Figure 2).

## 4. Discussion

The results of the present study showed that following acquisition trials, which led to a significant increase in learning and the probe test confirming memory retention in Barnes maze test, there were no substantial changes in the strength of Schaffer collaterals-CA1 synapses in the dorsal hippocampus. Field potential recordings before and after the Barnes maze test revealed that this hippocampal-dependent spatial learning task did not significantly change synaptic efficacy in the CA1 area of the dorsal hippocampus.

To evaluate the synaptic strength, we recorded the stratum radiatum of the CA1 region. In this area, we could directly record the changes in synaptic activity as this region was the sink of synaptic current, and measuring the slope or amplitude of the recorded fEPSPs was a good criterion of synaptic activity in this region. Besides, considering the critical role of the dorsal hippocampal CA1 area in spatial learning (Eichenbaum, 1999; Morris et al., 1982), this region was a suitable area for recording.

Learning-induced synaptic plasticity has been shown in the dentate gyrus region of the hippocampus. It has been reported that the slope of fEPSP increases in classical conditioning (paired tone and foot shock) (Doyere et al., 1995) and foot-shock unconditional escape in twoway shuttle-box avoidance (Matthies, Ruethrich, Ott, Matthies, & Matthies, 1986). In a previous study, Nomoto et al. observed 40% depression in population spike amplitude, 20% depression in molecular layer fEPSP slope, and only 7% increase in granular cell fEPSP slope monitored throughout an appetitively-motivated operant paradigm in freely moving rats (Nomoto, Yamamoto, Tomioka, & Nomoto, 2012). Learning-induced changes in synaptic efficacy in the dentate gyrus (either potentiation or depression) have also been reported in other studies (Aiba et al., 1994; Andersen, Bliss, & Skrede, 1971; Doyere et al., 1995; Skelton et al., 1987; Weisz et al., 1984).

Although many studies have investigated the learning-related synaptic efficacy changes in the dentate gyrus, few studies demonstrate changes in synaptic efficacy during learning in the CA1 region. In line with our observation (no significant changes in synaptic strength before and after learning and memory), Jonathan et al. recorded field potentials from CA1 stratum radiatum of the dorsal hippocampus during inhibitory avoidance task via 8 implanted electrodes in freely moving rats. They showed that although inhibitory avoidance task caused the delivery of AMPA receptors to hippocampal synaptoneurosomes and phosphorylation of hippocampal glutamate receptor, there was no significant difference in the average responses across all recorded channels before and after training. However, the variance between recorded channels increased significantly (Whitlock et al., 2006). Consistent with the present study, some genetic manipulations that disrupt LTP do not impair some forms of hippocampal-dependent memory (Zamanillo et al., 1999). Also, manipulations that do not alter hippocampal LTP may disrupt spatial learning (Shimshek et al., 2006).

The lack of changes in synaptic plasticity during learning in our study (and perhaps similar reports) may refer to technical problems. Given that the synapses involved in the storage of acquired information are few and may distribute in different neurons in the CA1 area (McNaughton & Morris, 1987), field potential recording may be an inefficient technique to detect synaptic modification potentially responsible for particular learning behavior. Therefore, learning-related changes in synaptic plasticity may be masked by an unrelated event to learning. In addition, in the rat hippocampus, the different kinds of spatial learning paradigm may induce the occurrence of plasticity in the different synapses of the trisynaptic circuit of the hippocampus (Hagena & Manahan-Vaughan, 2011; Kemp & Manahan-Vaughan, 2008; Manahan-Vaughan & Braunewell, 1999), i.e., the learning-induced plasticity may occur in the early synapses of the trisynaptic circuit (perforant-path to dentate gyrus or mossy fibers to CA3 synapses) that have not been monitored in our study.

Moreover, both LTP and LTD work together to encode the different aspects of spatial information (Hagena & Manahan-Vaughan, 2011; Kemp & Manahan-Vaughan, 2007), and because field potentials represent the summation and an average of these phenomena, this may lead to negation of each other. Therefore, no changes may be observed in the fEPSP parameters before and after learning. Similarly, it can be postulated that the synaptic potentiation may occur in both glutamatergic and GABAergic synapses, and excitatory and inhibitory synapses neutralize the function of each other. However, there was no difference in the paired-pulse index before and after the Barnes maze test. As the paired-pulse index at 30 ms inter-pulse interval is a sign of GABAA receptor activity (Adamec, McNaughton, Racine, & Livingston, 1981; Tuff, Racine, & Adamec, 1983), it indicates that the activity of GABAergic interneurons has not changed and the latter possibility may not happen.

One more probability is related to the hippocampal neuronal circuits. Some evidence suggests that in the CA1 region, behavioral activity alone is insufficient to trigger the synaptic changes that arise from the acquisition of novel spatial features, as the activated exploration of the featureless empty environment does not elicit persistent plasticity (Kemp & Manahan-Vaughan, 2004).

To sum up, our study did not support the idea that plasticity in Schaffer collaterals-CA1 synapses of the dorsal hippocampus is triggered by spatial learning events. However, the precise knowledge about the network of participating neurons behaves in both learning and non-learning tasks, and using more advanced methodological tools may help uncover the relationship between synaptic plasticity and learning and memory.

## Ethical Considerations

### Compliance with ethical guidelines

All ethical principles were considered in this article.
